# Multivalent Aptamer Approach: Designs, Strategies, and Applications

**DOI:** 10.3390/mi13030436

**Published:** 2022-03-12

**Authors:** Zhong Wang, Xiuying Yang, Nicholas Zhou Lee, Xudong Cao

**Affiliations:** 1 Ottawa-Carleton Institute of Biomedical Engineering, University of Ottawa, Ottawa, ON K1N 6N5, Canada; zwang480@uottawa.ca; 2 Hainan Institute of Science and Technology, Haikou 571125, China; xiuying.yang.hk@outlook.com; 3 Department of Microbiology and Immunology, McGill University, Montréal, QC H3A 0G4, Canada; nicholas.lee3@mail.mcgill.ca; 4 Department of Chemical and Biological Engineering, University of Ottawa, Ottawa, ON K1N 6N5, Canada

**Keywords:** aptamers, detection, multivalence, avidity, antibodies, biosensors

## Abstract

Aptamers are short and single-stranded DNA or RNA molecules with highly programmable structures that give them the ability to interact specifically with a large variety of targets, including proteins, cells, and small molecules. Multivalent aptamers refer to molecular constructs that combine two or more identical or different types of aptamers. Multivalency increases the avidity of aptamers, a particularly advantageous feature that allows for significantly increased binding affinities in comparison with aptamer monomers. Another advantage of multivalency is increased aptamer stabilities that confer improved performances under physiological conditions for various applications in clinical settings. The current study aims to review the most recent developments in multivalent aptamer research. The review will first discuss structures of multivalent aptamers. This is followed by detailed discussions on design strategies of multivalent aptamer approaches. Finally, recent developments of the multivalent aptamer approach in biosensing and biomedical applications are highlighted.

## 1. Introduction

The concept of multivalency is intrinsic to nature. For instance, marine mussels use multiple byssal threads to ensure attachment to rock surfaces, and octopus appendages make use of numerous suckers to efficiently capture preys. Likewise, multivalent interactions play a vital role in biological systems, down to the cellular level. Examples of biological processes utilizing multivalence include viral entry, cell–cell interaction, host–pathogen interaction, and immune synapse formation [1]. In each of these processes, multiple functional units work synergistically to improve overall performance. By taking advantage of the working principle of multivalency, researchers have applied this strategy in many molecular-recognition-based applications, such as targeted drug delivery [2,3], pathogen inhibition [4,5], cell capture and downstream analysis [6,7], detection [8,9], and in vivo imaging [10,11]. To this end, multimeric nucleic acids [12], antibodies [13,14,15], and aptamers have all been used to enhance functional performance in comparison to their monomeric counterparts.

Among the multivalency-based strategies mentioned above, the multivalent aptamer approach has attracted the most attention in recent years. Aptamers are short single-stranded DNA (ssDNA) or RNA molecules that bind to targets via specific structures [16] and possess advantages over other binding mechanisms, such as antibody-antigen binding. These short aptamer oligonucleotides are artificially selected from a process called Systematic Evolution of Ligands by EXponential Enrichment (SELEX) [17]. Aptamers can be selected to target ligands that are extremely small, highly toxic, or non-immunogenic, characteristics that are not readily achievable with conventional antibodies. In addition, thanks to large-scale oligonucleotide synthesis, the production of aptamers is significantly more cost-effective and accessible than that of antibodies.

In general, multivalent aptamer approaches aim to achieve enhanced binding avidities toward their target ligands. The avidity of an aptamer describes the overall affinity of the interactions between an aptamer and its target; by linking two or more aptamer units, multivalent aptamers have been shown to work cooperatively to increase the avidity of interactions [18,19]. Higher local aptamer concentration leads to a higher chance of interaction between aptamers and cell-surface ligands. In addition, after the initial binding of an aptamer with its ligand, nearby aptamers can be readily recruited to facilitate additional binding, thereby leading to a higher avidity. Physiological conditions present another challenge for aptamers; in their monomeric form, aptamers have been shown to be rapidly degraded by nuclease, a major drawback limiting their in vivo applications [20]. However, studies have demonstrated that conjugating multiple aptamers onto nanoparticles can successfully provide resistance to enzymatic degradation and increase nucleic acid stabilities due to increased local salt concentrations on the surfaces of the nanoparticles [21,22].

The current review focuses on the structures and design principles of multivalent aptamer approaches developed in recent years. To this end, multivalent aptamers are first divided into several categories based on their structures. This will set the stage for detailed discussions on the design principles and strategy highlights of multivalent aptamers. Subsequently, applications based on recently published studies related to multivalent aptamers are reviewed, followed by a brief discussion of current challenges and future research directions.

## 2. Multivalent Aptamer Structures

**Figure 1:**
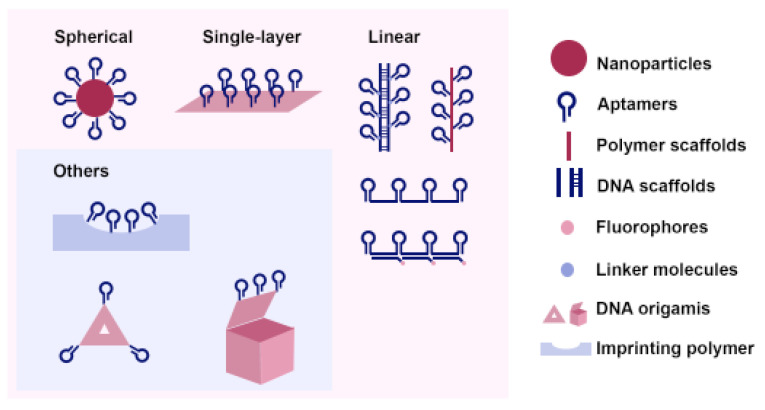
Schematic drawing of various structures of multivalent aptamer structures.

Based on their structures, multivalent aptamers can be generally divided into three categories: spherical [23,24], single-layer [25,26], and linear [27,28] (shown in Figure 1); other varieties of structures are also shown in Figure 1, and they are discussed in Section 2.4. In general, aptamers of interest are composed of three parts: a head-group moiety for attachment, a spacer, and an outward-extending capturing unit. While conjugating aptamers, one must tailor the conjugation method to the surface chemistry of each material. For different immobilization strategies, interested readers are encouraged to refer to recent review papers for details [29,30].

### 2.1. Spherical Multivalent Aptamers

For spherical multivalent aptamers, the aptamers are connected onto a hollow or solid core particle in an organized and radial polyvalent arrangement [31]. The core particles can be gold [32], silver [33], metal oxides [34,35,36], silica [37,38], polymers [39], or DNA self-assemblies [40]. Spherical carriers allow the greatest directional extension of aptamers into sample solutions, resulting in enhanced contact surface areas and thus better aptamer–target interactions. Furthermore, additional plasmonic [41], magnetic [24], or luminescent [42] properties of the particles can also offer added advantages for applications such as imaging and detections.

### 2.2. Single-Layer Multivalent Aptamers

After the first ground-breaking study on the two-dimensional (2D) material graphene was published in 2004 [43], a new chapter has started in the history of material science. The atomic thickness of 2D materials is the origin of their unique physical and chemical properties, which are considerably different from those of three-dimensional (3D) structures. For example, their unique optical properties make them a promising tool for photothermal therapy [44], detection, and imaging applications [45]. Moreover, the large surface-to-volume ratio of 2D materials also allows for increased loading of therapeutic agents [46,47]. Typical examples of 2D materials are graphene, transition metal dichalcogenides (e.g., molybdenum disulfide, MoS_2_), and phosphorene.

Besides 2D materials, conjugating aptamers onto DNA origami nanosheets can also achieve monolayer aptamer configuration. This type of aptamer has been used as a delivery vehicle for drugs [48,49], antisense oligonucleotides [48], antimicrobial lysozymes [50], and RNase A [51]. In addition to possessing high loading capacities and programmable structures, DNA origami-conjugated aptamers have also been shown to have good biocompatibilities. Additionally, the angstrom level precision of DNA origami scaffolds and their excellent spatial addressability created the possibility to realize single-molecule-level biosensing [25,52].

### 2.3. Linear Multivalent Aptamers

Conjugating aptamers onto linear scaffolds—either flexible or rigid—allows aptamers to be arranged into linear configurations. The linear scaffolds can be made of polymers [53] or nucleic acids, such as ssDNA [54,55], double stranded DNA (dsDNA) [56,57,58], and DNA nanotubes [59,60]. The physical properties of each scaffold type confer specific advantages to their resultant linear constructs. For flexible scaffolds such as ssDNA and polymers, the scaffold chains can easily adapt to the surface topography of targets, while the multivalent aptamers work synergistically, thus enhancing binding avidities [53]. Linear multivalent aptamers with flexible scaffolds are commonly used for therapeutic [4,61] or detection purposes [23,53,55]. Because of these long and flexible aptamer chains, the linear multivalent aptamers can reach tens of micrometers into the sample solutions, providing sufficient interactions with targets for more enhanced target capturing [55]. In addition, these flexible multivalent aptamer chains have been shown to wrap around their targets, resulting in even higher target-capturing efficacies [62]. In contrast, rigid scaffolds endow the aptamers with more precise arrangements and orientations. Typical rigid scaffolds include DNA nanowires and nanotubes; these rigid scaffolds are also commonly used as drug-loading sites for therapeutic agents [28,63].

### 2.4. Other Multivalent Aptamer Structures

Multivalent aptamer strategies can also be found in other configurations. For example, in an attempt to increase the avidity of molecularly imprinted polymers (MIPs), the multivalent aptamer approach has been used [64,65]. After removal of the template molecules, the polymers are left with binding sites and shapes that are complementary to the original template molecules. This MIP approach will likely find wider applications in molecular-recognition-based sensing and detection, especially if it is combined with multivalent aptamers to further enhance binding avidity [66,67]. In addition, DNA origamis, such as nanoboxes [68,69], nanorobots [60], and tetrahedral [70,71] and triangular origamis [72,73], can also be used to construct nanostructures that feature multivalent aptamers, as summarized in Figure 1.

## 3. Design Strategies

**Figure 2:**
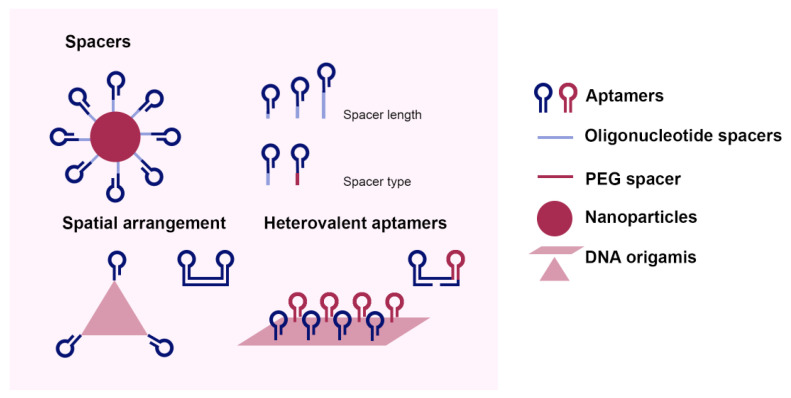
Schematic illustration of designs of multivalent aptamers. The optimization strategies include adding different lengths or types of spacer motifs, using a mixture of two or more different types of aptamers, and applying spatial arrangements of aptamers tailored for the geometries of target ligands and binding pockets.

The design of multivalent aptamer composites combines multiple identical or different aptamers with structural and other additional elements. The structural elements can be either a polymer (e.g., poly(ethylene glycol), PEG) or a non-specific nucleotide sequence (e.g., homo-polymeric oligonucleotides, such as oligo-T, with different lengths [4,54,55,74]). These structural elements are designed to maintain intra- and inter-molecular space that allow for independent activities of functional elements. Additional elements include functional nucleotide sequences, such as DNAzyme, small interfering RNA (siRNA), or signaling probes. In order to achieve optimal performances, other factors are also considered, such as spacers, heterovalent aptamers, and spatial arrangements of the aptamers, as shown in Figure 2. These factors will be discussed individually in the following sections. By applying one or combinations of these strategies, the efficiency of multivalent aptamer composites can be further enhanced. For example, by combining spacer designs with heterovalent aptamers, Lao and coworkers [75] demonstrated a hundred- to a thousand-fold enhancement of microarray sensitivity compared to single-type aptamers without any spacers.

### 3.1. Spacers

Spacers refer to short-strand polymers or oligonucleotides between the recognition domains of aptamers and the conjugated surface that they are attached to. Although multivalent aptamer strategies can enhance avidities compared to free aptamer monomers even without added structural elements [76], avidities can be further improved by employing properly designed spacers. This is because these spacers can either offer enhanced abilities for surface-tethered aptamers to properly bind to their targets or prevent aptamer units from forming unexpected secondary structures.

In general, surface-tethered aptamers with spacers show better performance when compared with those without. With properly selected lengths and materials, spacers can increase the avidities of multivalent constructs by overcoming steric hindrances due to adjacent aptamers. The most common spacers are PEG [77,78]; alkyl [78,79]; ssDNAs, such as oligo-T and oligo-A [78,80,81]; and dsDNA [82]. Among these different types of spacers, oligo-T spacers are perhaps the most commonly used [75]; therefore, in this section, we focus on oligo-T spacers.

Spacer length is an important consideration in spacer design. A spacer with properly designed length should both support the aptamer sequence and allow the aptamers to stand out from the conjugated surface or backfilling molecules [83]. Surface-immobilized aptamers with no or short spacer lengths experience impairments in freedom motions [84], while long spacer lengths can be too long to ensure correct folding of either the aptamers [81] or the spacers themselves [85] for correct secondary structures. For instance, Qin and coworkers [81] demonstrated the importance of spacer length by conjugating sgc8 aptamers onto poly(amidoamine) (PAMAM) dendrimers via oligo-Ts of lengths from 2 to 20 nucleotides for capturing circulating tumor cells (CTCs). The researchers observed that excessively long spacers resulted in unfavorable alterations in aptamer secondary structures, thereby negatively impacting the capturing performance. In addition, Edwards and coworkers [86] reported that an increase in oligo-T spacer length resulted in decreasing number of aptamers immobilized onto capturing surfaces. Furthermore, the researchers also reported that increasing oligonucleotide spacer lengths also caused increasing electrostatic repulsion, which, in turn, resulted in stronger electric-charge barriers that eventually inhibited additional aptamers to be immobilized onto the capturing surfaces [86].

### 3.2. Heterovalent Aptamers

Heterovalent aptamer strategies, also known as aptamer cocktails, use two or more types of aptamers that target different moieties of the target. In comparison with single type of aptamer, heterovalent aptamer approaches have been shown to result in the cooperative enhancement of overall aptamer performances. For example, an 18-fold improvement of limit of detection (LOD) was observed using an aptamer cocktail-modified electrode (three different aptamers targeting different moieties on the surface of *E. coli* cells) compared to an electrode modified with a single-type aptamer [87].

**Figure 3:**
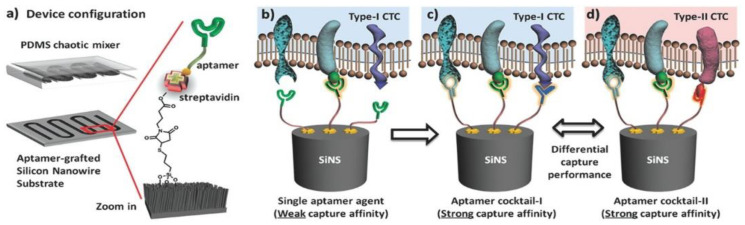
Schematic description of aptamer-cocktail-based microfluidic approach for CTC detection. (**a**) A microfluidic device is composed of an aptamer-conjugated silicon nanowire and a PDMS chaotic mixer. (**b**) When a single type aptamer is incorporated, the capturing affinity of the device is relatively weak due to the lack of synergistic binding. (**c**) By using an aptamer cocktail, the synergistic effects among individual aptamers lead to an enhanced capturing affinity. (**d**) Different aptamer cocktails are expected to have differential capture performance for CTC subpopulation recognition [89].

The aptamer-cocktail strategies have shown exciting potentials in CTC detections. Traditionally, CTC detections face issues such as low CTC occurrences in clinical samples and difficulties in targeting appropriate CTC biomarkers, since the presence of these surface markers is known to be non-uniform between individual patients [18]. To overcome these obstacles, one can target different arrays of CTC biomarkers simultaneously to both increase binding avidities and prevent the off-target effects due to the reduced or loss of certain surface biomarker expressions on the CTCs [88]. For example, Lin et al. [18] developed a dual-aptamer-tethered network system that allowed DNA-triggered reversible isolation-and-release of CTCs by tethering Sgc8c and Sgc4f aptamers onto a ssDNA scaffold. This dual-type aptamer network system showed significantly higher capturing efficiency compared to a single-type aptamer network. Similarly, Zhao et al. [89] combined the aptamer-cocktail strategy and poly(dimethylsiloxane) (PDMS) microfluidic detection platform to detect CTCs, as shown in Figure 3. The researchers showed that the aptamer cocktail exhibited a synergetic effect in target capturing and that the aptamer-cocktail approach demonstrated a higher overall capturing efficiency when compared with the single-type aptamer approach.

**Figure 4:**
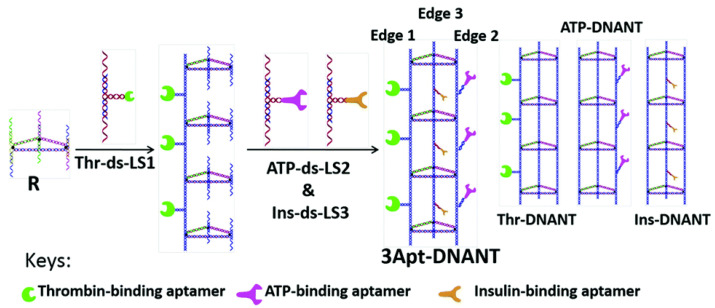
Schematic drawing of constructions of 3 aptamer-DNANTs and schematic representation of thrombin-DNANT, ATP-DNANT, and insulin-DNANT [91].

Another way of utilizing heterovalent aptamers is multiplex detection, which allows for the simultaneous detection of more than one target. Multiplex detections significantly reduce sample size and runtime required for detection. For example, by combining aptamer-conjugated gold nanoparticles (AuNPs) with magnetic graphene nanosheets, Dou and coworkers [90] developed a sensitive electrochemical platform for detecting CTCs in blood samples. Both Sgc8 and Td05 aptamers were incorporated to target two types of CTCs: CCRF-CEM cells and Ramos cells, respectively. This platform demonstrated successful detection of rare CTCs in clinical blood samples. In another interesting study, Dai et al. [91] developed an optical multiplex system based on a 3D triangular-shaped DNA nanotube (DNANT), as shown in Figure 4. As a proof-of-the-concept, three different aptamers to individually target thrombin, adenosine triphosphate (ATP), and insulin were conjugated onto three edges of the nanotube. This work demonstrated that the aptamer-conjugated DNA nanotube was able to achieve multiple binding activities that also generated discriminative signals for multiple targets in one detection system. The authors further suggested that by simply altering the design of the scaffold from triangular to square, pentagonal, and hexagonal nanotubes, it would be possible to simultaneously detect four, five, or even six targets.

### 3.3. Spatial Arrangements

The spatial arrangements of aptamers or their positioning relative to binding motif alignments on the target surfaces have recently become an interesting field in the study of multivalent aptamers. The optimal arrangements of aptamers—characterized by the distance and geometry of surface ligands or binding pockets—is believed to be more important than high local aptamer concentrations [5,61], since placing aptamers at an optimal distance and position can result in increased avidity [92].

**Figure 5:**
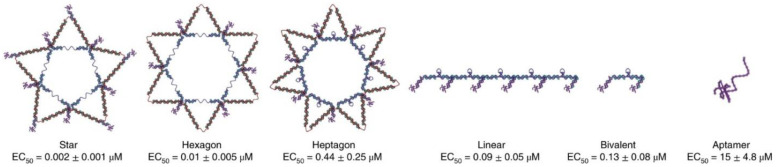
A schematic representing the inhibitory nanostructures for dengue virus with their corresponding mean EC50 (half maximal effective concentration) values. The hexagonal spacing resulted in reduced detection compared to the star-shaped spacing, despite its being the correct spacing. The schematic for the star and hexagon shows an unzipped hairpin region because potent pattern matching occurs. Other scaffolds represent the hairpins as stem–loop structures to indicate a lack of potent pattern matching ([5]).

Spatial arrangements can be achieved by either altering the distance between two adjacent aptamers [92,93] or positioning aptamers in a desired geometry [5,94]. Distance arrangements can be achieved by positioning aptamers onto rigid scaffolds (i.e., dsDNA, dsRNA [95], or DNA origami [96]). By conjugating two different anti-thrombin aptamers onto multi-helix DNA tiles and DNA origami scaffolds at various distances, Rinker et al. demonstrated that placing two different thrombin binding aptamers at an optimal distance on a rigid DNA tile nanostructure could lead to significant improvements in binding affinity, with results suggesting an estimated 50-fold enhancement in binding strength compared to aptamer monomer approach [97]. Using rigid scaffolds for spatial arrangement is particularly effective when the arrangements of binding moieties on the target surface can be perfectly aligned with those of the aptamers. However, it should be noted that even minor structural mismatches could result in suboptimal binding, due to unfavorable steric interactions in such cases [98]. In an elegant study, Kwon et al. [5] developed a star-shaped multivalent aptamer-conjugated DNA architecture by using tile-based DNA assemblies. In these star-shaped DNA nanoconstructs, each dsDNA intra-molecular spacer between the adjacent aptamers contained a hairpin structure, which could be unzipped and stretched to fine tune in order to fit the distance between adjacent surface ligands. This spatial arrangement of multivalent aptamers enhanced binding to their targets while reducing off-target binding through pattern matching. Significantly, the researchers also showed that aptamers with a mismatched geometric arrangement displayed an unexpected lower affinity to their targets, suggesting that specific bindings between aptamers and their targets are dependent on not only individual aptamer–ligand interactions but also geometry matching and pattern recognition. As shown in Figure 5, a heptagon-shaped complex showed a poor detection performance despite correct spacing and a higher level of valency, thus strongly suggesting the importance of geometric positioning [5].

## 4. Biosensing and Biomedical Applications

### 4.1. Biosensors

Aptamer–target interaction is an important recognition mechanism in biosensing applications. In particular, multivalent aptamers allow elevated local concentrations of aptamers and increased binding avidities between the capturing motif and the target. As a result, the multivalent strategy has been widely used in many large-sized target detections, such as human cells [6,18,42,67,99] and bacterial cells [87,100,101,102] in whole-cell assays. In addition, the multivalent strategy has also been used to detect smaller sized targets, such as extracellular vehicles [54,103] and small molecules [104]. In comparison with antibodies, aptamers allow readily and reversible capturing of targets for further analysis and characterizations [105].

Linear aptamers and spherical aptamers (see Figure 1) are the most frequently used aptamer forms in detection applications; to further increase multivalency, the combinations of the two forms have also been reported [24,100]. For instance, Chen et al. [24] proposed a NanoOctopus to capture CTCs in blood samples. To achieve this, the researchers conjugated multivalent aptamer chains onto the surfaces of magnetic nanoparticles (i.e., NanoOctopus), and they showed that the multivalent NanoOctopus significantly increased binding avidity to their targets. Subsequently, the captured cells were released for further analysis. Similarly, DNA nanochains that contain multiple copies of aptamers have also been used [55,62,100]. Inspired by climbing plants, Liu et al. [53] developed a multivalent aptamer nanoclimber (MANC)-functionalized PDMS microfluidic device for minimal residual disease testing. This work demonstrated a 57.5-fold increase in binding affinity in comparison with a mono-aptamer approach when the MANCs was used to capture target cells in human plasma samples, suggesting that the MANCs are clinically applicable to assay minimal residual disease in peripheral blood. The captured cells were then released by using complementary DNA displacement or nuclease digestion for downstream analysis.

Spherical multivalent aptamers have also been used in microfluidic devices [6,19,23,106] to increase detection performances of the microfluidic devices. Inspired by octopus tentacles, Song et al. [23] conjugated SYL3C aptamers onto AuNPs that were subsequently attached to the inner surfaces of a micropillar-decorated microfluidic channel. The researchers showed that approximately 250 SYL3C aptamers were attached onto each AuNP and that the detection platform was able to detect CTCs directly in blood samples without any sample pretreatments. In addition, the authors further demonstrated that the multivalent aptamer approach improved the binding efficiency by 100-fold and that the capturing efficiency was enhanced by more than 300% in blood samples when compared with aptamer-monomer-modified microfluidic devices. The captured target cells were released by using a thiol-exchange reaction that was shown to be of high efficiency and maintained high cell viability. Alternately, polymeric dendrimers, such as PAMAM, have also been used to construct spherical multivalent aptamers structures [101]. Jiang and coworkers [100] developed a fluorescent microfluidic sensing platform for detecting *E. coli* O157:H7, using rolling circle amplification (RCA) chain-conjugated PAMAM dendrimers as an inner-channel surface modification. In this work, the PDMS microfluidic channel was modified with PAMAM dendrimers that were subsequently conjugated with capturing long ssDNA chains containing hundreds of repeating aptamer units. The resultant channel modified by multivalent aptamer chains captured approximately 5-fold more target cells compared to a channel modified by aptamer monomers. More importantly, this multivalency-driven improvement was observed to be more significant under higher flow rates, suggesting that this multivalent strategy can be a potential solution for achieving higher throughput in microfluidic devices while maintaining sufficient capturing efficiencies.

### 4.2. Biomedical Applications

Targeted therapeutic-treatment deliveries generally require specific interactions between cell-surface biomarkers and recognition elements [107]. This specific recognition mechanism enables accumulation of therapeutic agents in pathological sites while reducing systemic toxicity [63]. Aptamer-mediated drug-delivery systems take advantage of the enhanced avidities, bio-stability, and intracellular uptake to their targets via multimerization. For instance, Wang et al. [108] used a DNAzyme-driven DNA nanosponge with multivalent aptamers for targeted delivery and controlled release of doxorubicin. In addition, carriers such as aptamer-conjugated DNA self-assemblies can also enable targeted delivery of gene therapies, such as the delivery of siRNA [56,109], DNAzyme [110], and Cas9/sgRNA (small guide RNA) [111,112]. It is conceivable that co-deliveries of more than one type of therapeutic agents are also possible.

Another important area of in vivo multivalent aptamer applications is focused on cancer treatment. Over the past decades, many aptamers that specifically target cancer cells have been successfully selected, including AS1411 (nucleolin-targeted) [113,114], MUC1 (MUC1-targeted) [115], and Sgc8 (protein tyrosine kinase-7-targeted) [38], among others [39,74]. However, aptamers have not yet been widely used in in vivo cancer treatments. This is likely, in part, due to their poor performances under physiological conditions. For example, aptamers are known to be vulnerable to nuclease degradations [116]; in addition, conditions such as temperature [61,117], pH [118], and shear stress [105,119] have also been shown to affect the abilities of aptamers to bind to their targets. As a promising solution to these challenges, multimerizing aptamers have been used to mitigate in vivo instabilities associated with aptamers, since multimerizing aptamers have been shown to increases steric hindrance, thereby concealing specific cleavage sites from nuclease access [120]. For example, Li et al. [28] prepared a self-assembled DNA nanocentipede as a multivalent drug carrier by hybridization chain reaction (HCR). This drug carrier remained stable after treatment with Exonuclease III for 2 h, demonstrating significantly increased resistance to degradation.

In addition, multimerizing aptamers for in vivo applications can enhance the internalization of the delivery vehicle by target cells. Endocytosis is of interest in understanding the mechanisms that guide the entry of nanomaterials into target cells, which is particularly meaningful for in vivo applications [121]. The multivalent effect has been shown to enhance cellular uptake via receptor-mediated endocytosis by improving the local concentration of aptamers [56,63,122]. To demonstrate this concept, Kang et al. [123] immobilized multivalent aptamers onto a DNA or a DNA/RNA hybrid structure to build a targeted drug-delivery system. For comparison, the researchers separately conjugated dual-type aptamers (AS1411 and MUC-1) and single-type aptamers onto multiple scaffolds of different levels of valency (i.e., monomers, pentamers, nonamers, or RCA generated multivalent scaffolds). The RCA-generated multivalent dual-type aptamer system outperformed monomer aptamer systems with a 2.9-fold higher intracellular uptake in serum-free media.

### 4.3. Summary of Recent Research

In addition to the applications discussed above, recently published studies on multivalent aptamers are summarized Table 1. The table lists applications, aptamers, targets, conjugated materials, structures, and demonstrated advantages of aptamer multivalency; interested readers are encouraged to refer to the original publications for more details.

**Table 1:** A summary of recently published multivalent-aptamers-related papers.

Application	Aptamer	Conjugated Material	Target	Functional Component	Spacer	Advantages of Multivalency	Reference
**Spherical multivalent aptamer**							
Drug delivery	SZTI01 (DNA)	RCA generated spherical DNA self-assembly	Prostate cancer membrane antigen (PCMA)-positive C4-2 cells (PCMA)	Drug-loading sites; doxorubicin	pH-sensitive oligo-T	Very stable in physiological environment; high specificity	[74]
Drug delivery	5TR1 (DNA)	Poly(lactic-co-glycolic acid) (PGLA) dendrimer; chitosan	MCF7 cancer cells (MUC1 receptor)	Drug-loading sites; epirubicin	N/A	High affinity and specificity; good internalization	[39]
Drug delivery	S2.2 (DNA)	Lipid-capped polymer nanoparticle	Cancer cell	Drug-loading sites; vinorelbine	PEG2000	Significantly higher uptake efficacy; enhanced cell targeting efficiency with increased density of the S2.2 aptamer	[124]
Drug delivery	AS1411 (DNA)	Chitosan-silica nanoparticle	SKOV-3 ovarian cancer cell	Drug-loading sites; epigallocatechin gallate	N/A	Higher internalization efficacy	[125]
Imaging/Therapeutic	AS1411 (DNA)	AuNPs; Silver nanoclusters (AgNCs)	Cancer cell	Drug-loading sites; doxorubicin; DNA-templated silver nanoclusters (signaling, fluorescent)	Adenine-rich sequence	Increased the stability of DNA probe; better internalization	[114]
Detection and isolation	MUC1 (DNA)	Hybrid membrane; Fe_3_O_4_@SiO_2_ magnetic nanoparticle; Ag_2_S nanodots	CTC	Ag_2_S nanodots (signaling, near-infrared fluorescence signal)	DNA scaffold	Greatly enhanced the anti-interference from background; improve binding ability; good specificity	[42]
**Single-layer multivalent aptamers**							
Imaging	Sgc8 (DNA)	Manganese dioxide nanosheet	CCRF-CEM cells	MnO_2_ (signaling, contrast agent); fluorescent-labeled aptamer (signaling, fluorescence)	N/A	Target-specific binding and internalization; low background signal; high sensitivity	[126]
Chemo-photothermal therapy	AS1411 (DNA)	Black phosphorus nanosheet	PC3 cells	Doxorubicin; black phosphorus nanosheet (photothermal therapy)	N/A	Specific and high photothermal cytotoxicity for target cells	[26]
Drug delivery system	C2NP (DNA)	DNA origami	K299 cells (CD30 receptors)	Doxorubicin	N/A	Increased internalization; significantly increased aptamer bioactivity	[49]
**Linear multivalent aptamers**							
Detection	AS1411 (DNA)	RCA generated multivalent chain	B16 cells	Signaling molecular beacon (fluorescent)	18-bp dsDNA	Enhanced sensitivity; enhanced selectivity	[27]
Therapeutic	LS Aptamer (DNA)	RCA generated multivalent chain	Jurkat cells (L-selectin)	Aptamer (L-selectin inhibitor)	20 oligo-T	High avidity (103-fold higher affinity than L-selectin aptamer monomers); high specificity	[4]
Detection/cell manipulation	Split ZY11 (DNA)	RCA generated DNA chain	Human liver cancer	Signaling probe (fluorescent)	41 oligo-T; also served as signaling compartment by hybridization with signaling probe	Improved performance in avidity (a ~2.8-fold increase in signal-to-background-ratio); increased recognition; extended the tolerance range of temperature for target binding	[127]
Detection	SYL3C (DNA)	RCA generated multivalent aptamer network electrode interface	CTC (EpCAMs)	Anti-EpCAM antibody; horseradish peroxidase conjugated gold nanoparticle probe (signaling. electrochemical)	20 oligo-T	Greatly increased current response; reduced detection time	[55]
Drug delivery	AS1411 and MUC-1 (DNA)	DNA and DNA-RNA hybrid structures	MCF-7 cells	Drug-loading site	dsDNA or siRNA duplex	Increased intracellular uptake of dual aptamer systems (increase in aptamer valency led to higher intracellular uptake)	[123]
Detection	Zy1 (DNA)	HCR generated DNA self-assembly	Human liver cancer SMMC-7721 cells	Signaling branch (fluorescent)	10 oligo-T; HCR generated backbone	High stability in the presence of nuclease or in human serum; higher signal-to-background ratio compared with aptamer monomers; reduced dissociation constant (1/10) compared with Zy1 monomers	[128]
**Others**							
Drug delivery	Sgc8 (DNA)	DNA origami generated self-assembly (triangular)	Cancer cell/HeLa cell	Drug-loading sites; doxorubicin	N/A	Enhanced drug-delivery efficiency and therapeutic efficacy; decrease systemic toxicity; high stability in cell culture medium for 24 h	[73]
Targeted gene therapy	Sgc8 (DNA)	AAV2 vectors	CCRF-CEM cells (PTK7)	Gene for green fluorescent protein	DNA dendrimer	21-fold enhanced binding affinity and enhanced resistance against nuclease degradation	[129]
Immunotherapy	KK1B10 (DNA)	HCR generated DNA self-assembly-functionalized natural killer cells	K562 cells	Natural killer cells	HCR generated backbone	Greatly increased binding avidity compared to the aptamer monomers; good stability under physiological shear stress	[119]
Detection and capture	ZY-sls (DNA)	Cell imprinted hydrogel	SMC-7721 cells	N/A	Trifunctional cleavable crosslinker	Enhanced interaction between enhanced sites and target cells; high capture efficiency and selectivity	[67]

## 5. Challenges and Outlook

Multivalency is a strategy inspired by nature. In the last decade, multivalent aptamers have attracted a significant amount of interest as a promising solution to overcome the intrinsic challenges facing aptamer monomers by significantly improving avidity and stability. The rapid growth of research in material science further accelerates the ongoing development of multivalent aptamer-based devices. Encouraged by the significant advantages held by this approach, recent research is now focusing on developing constructs that excel in performance and possess potentials for use in real-life applications. However, there are many challenges that need to be addressed before the potential of the multivalency strategy can be fully realized.

Although the enhancement of local concentration is one of the advantages of multivalent aptamers, in some cases, it is not optimal to follow the idea of “the more, the better”, but rather, “less is more”. When designing an aptamer device, one must ensure the aptamers are properly positioned to expose their binding pockets to the greatest extent. In order to achieve this, different types of structural elements are often used, and the effects of these structural elements are critically important and warrant further investigations.

Spatial arrangements of aptamers is another important factor to consider in multivalent aptamer design. Proper spatial arrangements, optimal positioning, and correct orientation of aptamers can also significantly enhance overall avidities. As discussed, there has been some work performed to investigate the proper geometries between neighboring aptamers, with significantly higher avidities observed at certain geometric configurations. To properly configure the spatial arrangements of the aptamers in designing multivalent aptamer constructs, a deep understanding of target morphologies, ligand alignments, and aptamer–target interactions is necessary, and more research in this area is needed.

## 6. Conclusions

Multivalent aptamer-based applications have shown great potentials due to their enhanced performances in comparison with their monomer aptamer counterparts. By combining the design considerations of spacers, spatial arrangements, and the heterovalent effect, multivalent aptamer constructs have great potential for even more efficient functions and improved performance. Furthermore, by incorporating nanotechnologies, signaling molecules, and functional oligonucleotides into multivalent aptamer constructs, multifunctional smart devices that combine properties for applications in imaging, drug delivery, and diagnostics in real-world applications are perceivable in the near future.

## Data Availability

Not Applicable.
